# Perspectives on health examination for asylum seekers in relation to health literacy – focus group discussions with Arabic and Somali speaking participants

**DOI:** 10.1186/s12913-019-4484-4

**Published:** 2019-09-18

**Authors:** Josefin Wångdahl, Ragnar Westerling, Per Lytsy, Lena Mårtensson

**Affiliations:** 1 Department of Public Health and Caring Sciences, Uppsala University, Uppsala Science Park, Box 564, 751 22 Uppsala, Sweden; 2 Institution of Department of Neuroscience and Physiology/Occupational Therapy, University of Gothenburg, Box 455, 405 30 Göteborg, SE Sweden

**Keywords:** Health check-up, Health communication, Health literacy, Migrants, Qualitative research

## Abstract

**Background:**

Asylum seekers coming to most countries are offered a specific health examination. A previous study concluded that a considerable proportion of those taking part of it in Sweden had poor experiences of the communication in and the usefulness of this examination and had poor health literacy. The aim of this study was to explore in greater depth the experiences of the health examination for asylum seekers among Arabic- and Somali-speaking participants in Sweden. A secondary aim was to examine experiences and discuss findings using a health literacy framework.

**Methods:**

Seven focus group discussions were conducted with 28 Arabic and Somali speaking men and women that participated in a health examination for asylum seekers. Data were analyzed by latent content analysis.

**Results:**

One overarching theme - beneficial and detrimental - was found to represent the participants’ experiences of the health examination for asylum seekers. Three categories were identified that deal with those experiences. The category of “gives some good” describes the examination as something that “gives support and relief” and “cares on a personal level.” The category of “causes feelings of insecurity” describes the examination as something that “lacks clarity” and that “does not give protection.” The category “causes feelings of disappointment” views the examination as something that “does not fulfil the image of a health examination” and “does not focus on the individual level.”

**Conclusion:**

The health examination for asylum seekers was experienced as beneficial and detrimental at the same time. The feelings were influenced by the experiences of information and communication before, during and after the examination and on how health literate the organizations providing the HEA are. To achieve more satisfied participants, it is crucial that all organizations providing the HEA become health literate and person-centered.

**Electronic supplementary material:**

The online version of this article (10.1186/s12913-019-4484-4) contains supplementary material, which is available to authorized users.

## Background

In 2015, the world had about 3.2 million asylum seekers, i.e. individuals who seek international protection and whose refugee status is yet to be determined [1]. To identify poor health, secure the well-being of asylum seekers and guarantee the safety of the population in the host country, many countries conduct health examinations specifically assigned for asylum seekers (HEA) [2]. Another purpose of an HEA is to inform newcomers about the health system to assist in their access to healthcare [3–5]. In Sweden, the world’s third largest recipient of individual asylum applications in 2015 [1], asylum seekers are offered an HEA either before or after having received a residence permit as a seeker of asylum [5]. National guidelines exist about the content of the HEA [5]. It should be voluntary and free of charge. It should include a conversation about the participant’s past and present physical and mental health, including questions about infectious disease and immunization status. A physical examination and further medical investigations or tests may result from what comes up in the conversation. In addition, information should be given about asylum seekers’ rights to access required health and dental care. Thus, the HEA serves a dual purpose: improving and maintaining the health of the individual as well as attempting to prevent infections in society. However, adherence to the guidelines varies, including whether the participant meets with a doctor or a nurse during the HEA.

A report from 2014 shows that only about 40% of asylum seekers in Sweden participate in an HEA [6]. Factors such as different views on health, mistrust of healthcare and barriers related to information and communication may contribute to this relatively low proportion utilizing the HEA [7, 8]. These are all factors that can be affected by health literacy (HL). Data from our previous quantitative study regarding the experiences of HEA in Sweden show that a considerable proportion of participants had poor experiences of the communication in and the usefulness of the HEA as well as poor HL [9]. On the individual level, health literacy can be described in short as a person’s ability to find, understand, communicate, judge and use health information in order to improve and maintain health during the course of life [10]. At the system level, an organization can be defined as health literate if it supports persons in navigating in the healthcare system and understanding and using information and services to take care of their health [11]. In this study the integrated conceptual model of HL by Sorensen et al. [10] was used as a theoretical framework. It combines a conceptual model including different dimensions of HL with a logical model representing the impact of societal, personal and situational factors on HL, and connects HL to health outcomes.

To gain an in-depth understanding of the experiences of the HEA based on the results of the study mentioned above, and to discuss the possible role HL has in the context of HEA, more knowledge is needed about individuals’ subjective experiences of the HEA.

### Aim

The aim of this study was to explore, in greater depth, the experiences of the health examination for asylum seekers among Arabic- and Somali-speaking participants in Sweden. A secondary aim was to examine experiences and discuss findings using a health literacy framework.

## Methods

### Study design

The study had a qualitative design, using focus group discussions [12] as the method for data collection. The informal design of focus groups and the interaction between people in the discussions may support them in exploring and clarifying their views in ways that would not be accessible in one-on-one interviews [13]. Thus, this form of data collection stimulates new ideas and insight on the part of participants, allowing them to provide information that the researcher may not originally have considered [12]. Focus groups address the collective view, not the individual one [12], although individual experiences also emerge in the discussions [14]. The method is often used in cross-cultural research working with ethnic minorities, as the interpersonal communication may uncover shared knowledge in the form of sub-cultural values or norms [13].

Ethics approval was sought at the regional ethics committee. However, they deemed that the study design and data collection did not involve potential ethical conflicts, applicable according to Swedish law (Ethical Committee of Clinical Investigation in Uppsala, Dnr: 2013:446). Despite not needing a formal ethical approval, the project adhered to the standards of the Helsinki ethical principles in conducting the study.

### Participants and recruitment

Inclusion criteria were speaking Arabic or Somali fluently, having received a permanent resident permit as a seeker of asylum (i.e. people that have previously been asylum seekers but received a residential permit allowing them to stay in Sweden permanently) and having participated in an HEA in the last 3 years. The languages were chosen before the recruitment of participants based on statistics showing that these were the most common languages spoken by newly arrived refugees taking part in the HEA at the time of the planning of the study [15]. For ethical reasons, only those having received a residential permit were included. Otherwise potential participants were considered at risk of feeling obliged to participate because of a belief that participation would affect their chances of achieving permanent residential status.

In four cities, in different geographical regions of Sweden, a number of schools offering Swedish for immigrants (SFI) and centers offering civic orientation [16] were contacted and verbally informed about the study. Teachers then informed students and distributed information letters and interest registration forms in Somali and Arabic. Students were also invited to disseminate information about the study to others, i.e. purposive and snowball samplings were used [17]. The letter included information about the purpose of the study, that participation was voluntary, the possibility to withdraw at any time without explanation, and the confidentiality of the treatment and presentation of data. The registration form consisted of questions regarding participation in an HEA, country of birth, gender, age, and education level. The characteristics were collected to create homogeneous focus groups in order to minimize the risk of constructing focus groups that would inhibit participants from expressing themselves due to possible power relations. Participants with different gender and education level were divided into different groups. The characteristics were also used to secure heterogeneity in the overall study population in order to capture different experiences of the HEA [12], i.e. to ensure that participants with different country of birth, gender, age, education level and participation in HEAs at different locations were included. Those who volunteered to participate and had given contact details were then contacted and given practical information about their focus group discussion. The recruitment of participants was ended when saturation of data was achieved.

In total, 28 students consented to participate in the study. The number of people who refused to participate is unknown, because the SFI teachers who distributed the information and invitation letter did not record the number of persons who declined. Those who took part were divided into seven focus groups with three to five participants in each group; four had Arabic and three had Somali speaking participants. Zero to 9 years of education was classified as low, and 10 years or more as high. Difficulties in finding highly educated Somali women resulted in that no focus group was being held. All participants had received a permanent residential permit for reasons of asylum, most of them in the past 2 years. Altogether, the participants had taken part in HEAs in about 15 locations in different geographical regions in Sweden, most with an interpreter present. Detailed characteristics of the participants are presented in Table 1.

**Table 1:** Characteristics of participants in the study (*n* = 28)

Factor	N
Gender	
Men	16
Women	12
Age (years)^a^	
Range	24–67
18–29	6
30–39	8
40–49	5
50 or older	6
Educational level^b^	
No education/illiterate	3
1–6 years	5
7–9 years	4
10–12 years	7
More than 12 years	6
Country of birth	
Palestine	2
Jordania	1
Somalia	13
Syria	12
Native language	
Arabic	15
Somali	13
Years since participated in HEA	
< 1	16
1–2	11
> 2	1

^a^Three participants were between18 and 65; exact ages are missing. ^b^ Three participants had < 10 years of education; exact level is missing

### Study setting and data collection

The focus groups gathered in premises used by or in proximity to courses in Swedish for immigrants and civic orientation in four cities in Sweden. The study setting was chosen to make the informants feel relaxed and secure [12].

A guide [12] was developed by the authors according to the aim of the study and consisted of questions about experiences of one’s own HL in the context of health information in the HEA. All questions are presented in Additional file 1. The guide was translated into Arabic and pilot tested in the first focus group. The evaluation indicated that it should be kept in the original form. It was translated thereafter into Somali. Probing questions were used in order to gain a deeper understanding. To minimize the risk that participants would refrain from saying things out of politeness or a fear of authorities, verbal information was given on site that neither the moderator nor the researcher worked in Swedish healthcare or at the Swedish Migration Agency.

The focus group discussions were moderated by one Arabic- and two Somali-speaking women. The choice to use female moderators was based on recommendations to use few moderators [18] and recommendations by key people in the target groups who believed that a female moderator would make it easier for both women and men to speak more freely, in comparison to a male moderator. All moderators had academic degrees in public health or human rights and had cultural competence [19], i.e. spoke the same language and came from the same countries as most of their focus group participants. Two of the three had previous experience of moderating focus groups. Before moderating the discussions, they participated in a session including focus group methodology, ethics, and the purpose and background of the study. The first author took part in the focus groups as an observer and made field notes. Neither the moderators nor the researchers had a prior relationship with the participants. After each discussion, the moderator and the first author talked about the content, the group dynamics and what could be improved methodologically in the next focus group discussion. All focus group discussions were audio recorded, ran for about 1 hour, and took place in the period autumn 2014 - spring 2016.

The Arabic moderator transcribed the files recorded in Arabic directly into Swedish as she had qualified translation skills. An external translator with extensive experience of moderating focus groups and transcribing data, qualified translation skills and an appropriate academic degree was used for the recorded Somali discussions. A transcript protocol by McLellan [20], which describes how to write what was recorded in a structured way, was used when writing the transcripts. To check for interpreter bias [19], 10% of each transcript was checked for accuracy by independent bilingual persons. Some smaller differences were found but were not judged to affect the content.

### Analysis

The analysis was based on Graneheim and Lundman’s method for latent content analysis [21], which aims to find the latent meaning of the data. The analytical process is active and proceeds from a clinical-empirical to a more abstract level, which means it leaves the descriptive level. The analysis was carried out in several steps, in which two of the authors (JW and LM) took the most active part. Both are Swedish-born females without a migrant background with extensive knowledge and practical experience in the field of health promotion, HL and migration. Reflexivity was considered in the form of acknowledging preconceptions as a factor that could potentially influence the results during the analysis.

As the transcripts from the respective discussions were completed, the first author read them to form a first picture of the content and to be able to further develop the questions in the moderator’s guide. This first glance at the transcriptions gave an idea of when saturation of data was achieved. However, only when all the data had been collected and transcribed did the latent content analysis begin.

The transcripts were independently read in the first phase. This was followed by distinguishing, condensing and coding meaning units. When questions arose regarding ambiguities in texts from the transcripts, the moderator was contacted for a clarification. When all the relevant features of the data (i.e. data that related to the purpose of the study) had been coded, the codes were compared and discussed between the first and last authors in order to identify their latent content, i.e. the underlying meaning of the text [21]. Examples of the analytical process are given in Table 2.

**Table 2:** Examples of meaning units, condensed meaning units, codes, sub-categories and categories^a^

Meaning unit	Condensed meaning unit	Codes	Sub-category	Category
A2P3: They took information A2P1: They took information. They gave us none. A2P5: They didn’t either say about the results. We don’t know. A2P1: No information and not even advice. They information and asked what difficulties we had.	HEA did not give any information, it just took.	HEA takes more than it gives.	Does not focus on the individual	Causes feelings of disappointment

^a^First capital letter indicates focus group; second capital letter indicates participant

**Fig. 1:**
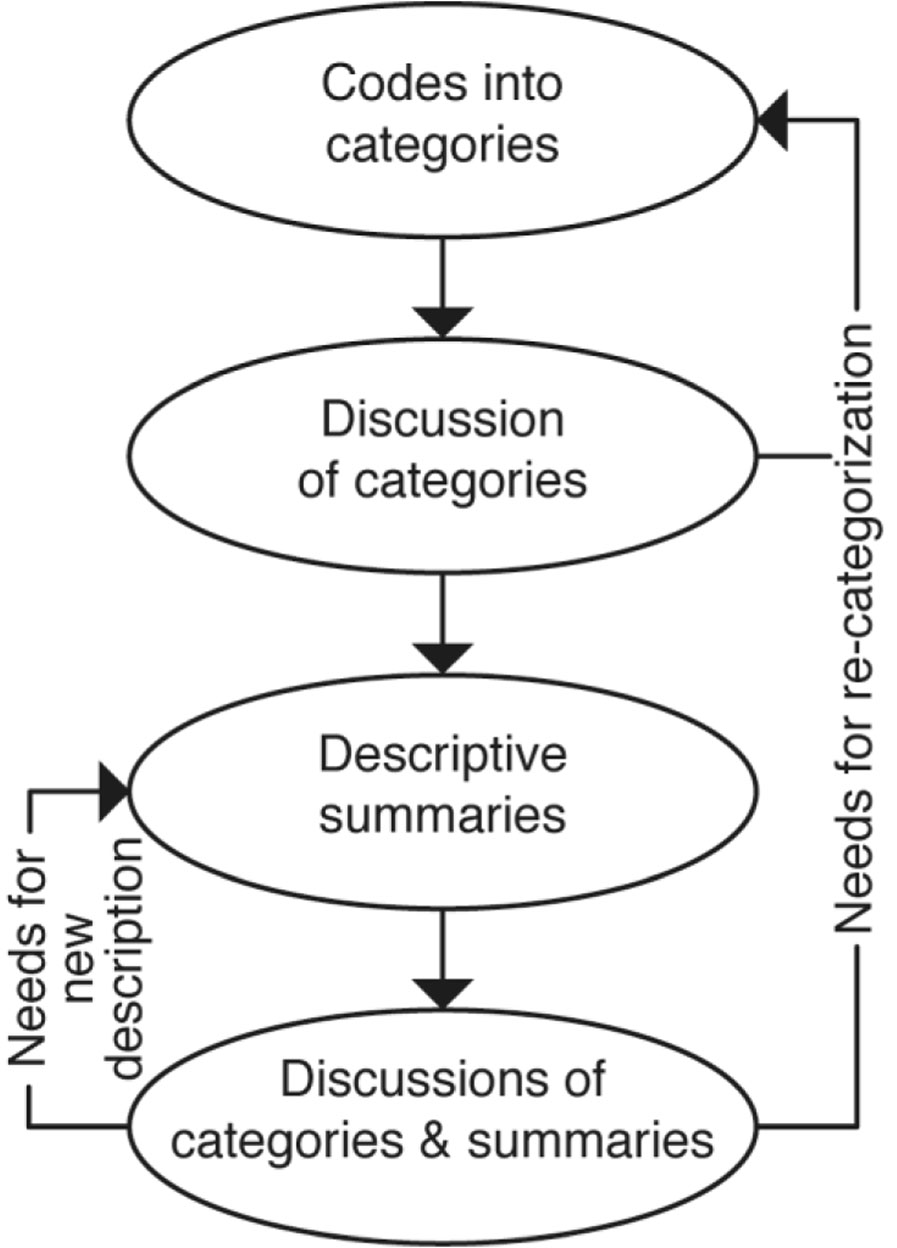
A flowchart of phase 2

Phase 2 began with sorting the codes into categories and sub-categories based on similarities and differences. Several preliminary versions, as well as relations between the categories, were tried and considered. This process resulted in rearrangement and the withdrawal of some categories and/or sub-categories and inclusion of some new ones, until all were distinctly separate. After this process, descriptions of their latent content were formulated. A constant exchange between raw data and the emerging categories ensured that the categories and sub-categories were based on the empirical experiences. The formulations were then repeatedly edited in order to further clarify and distinguish categories. A flowchart of phase 2 is presented in Fig. 1.

In the third phase, a common theme was formulated based on the latent content of the participants’ experiences as they were described in the categories. [21]. Finally, quotes from the focus group discussions were chosen to illustrate and validate each category, and were translated by a professional translator into English. Two of the moderators were asked to review the final results, checking the accuracy of the interpretations and trustworthiness of the results. Results and interpretations were judged to agree. We did not aim to investigate differences in findings by gender, origin of country or education level.

## Results

The participants’ experiences of the HEA are presented in one overarching theme, three categories and six sub-categories. The descriptions of the content are based on the discussions as a whole, the categories and the interpretation of the latent meaning, i.e. not explicitly the spoken words [21]. To emphasize the empirical foundation, samples of significant citations are provided after each category. The structure of the theme, categories and sub-categories is shown in Table 3.

**Table 3:** Ovraching theme, categories ad sub-categories

Theme	Beneficial and detrimental					
Category	Gives some good		Causes feelings of insecurity		Causes feelings of disappointment	
Sub-category	Gives support and relief	Cares on a personal level	Lacks clarity	Does not give protection	Does not fulfill the image of a health examination	Does not focus on the individual

### Beneficial and detrimental

This overarching theme describes contradictory experiences of an HEA, as it is viewed as both promoting health and being detrimental to health. The health-promoting experiences are described in the category “gives some good” and experiences that HEA might be detrimental to health are described in the categories “causes feelings of insecurity” and “causes feelings of disappointment.”

### Gives some good

This category describes the experiences of an HEA as something beneficial to the individual participating in it. The category comprises two sub-categories that describe different ways in which an HEA is experienced as giving good. Quotes from the focus groups describing the sub-categories are given in Table 4.

**Table 4:** Sub-categories and sample quotes from the focus groups^a^ describing the category “gives some good”

Category	Sub-category	Quotes
Gives some good	Gives support and relief	A4 (P1): It was good to know that I didn’t have the diseases that they tested for … A4 (P3): And I got assurance about my health. In general I was calmed about my health situation. S2 (P1): After a health examination, you get rid of a lot of worry that you’ve had and find out that everything is okay. S3 (P2): I found out that I had diabetes and then I got the right treatment. A3 (P3): They found two diseases and made me aware … And I followed them up when I was finished there … A2 (P5): I told them that I had a knee injury … and they sent me right away to get sessions with a physical therapist … S3(P2): … at the health examination that I took I found out also that I had diabetes. After that I got the right treatment.
	Cares on a personal level	S3 (P1): … they took good care of me … they welcomed me – showed me where to sit – they made me feel secure and showed me respect – and in terms of language, an interpreter was there. It didn’t matter if you could speak good Swedish or Somali, they had an interpreter there anyway. A4 (P1): They were cooperative and even asked me whether there was something I wanted to bring up … I think they met me in a good way. S1 (P1): … then I asked for a health examination, which they gave me.

^a^*A* Arabic speaking focus group, *S* Somali speaking focus group, *P* Participant

### Gives support or relief

This sub-category is based on the experience of being supported or relieved. One instance of being relieved by the HEA is receiving information about one’s health status. Test results that indicate good health can give relief, as they provide evidence that one is not a health risk to the new society. Other ways of experiencing relief are the HEA detecting a misdiagnosis brought from the home country or incorrect medications and that it does not introduce new diagnoses just to make money. Support is experienced by receiving help with health problems that are found, either directly at the examination or later at a referral to another service. Another way of being supported by the HEA is by receiving information about self-management and how living habits impact health.

### Cares on a personal level

This sub-category of the HEA as giving some good is based on the experiences of being cared for on a personal level. This is experienced by being encouraged to ask questions and talk about personal health issues, and by the fact that interpreters are hired to facilitate two-way communication and understanding. Being offered a more extensive examination and receiving information about personal health issues also contribute to the experiences of the HEA as giving some good. Receiving good personal treatment by friendly and understanding staff is experienced as creating feelings of being a person who is seen and welcomed to Sweden.

### Causes feelings of insecurity

This category describes the experiences of the HEA as being detrimental to health because it gives feelings of insecurity. The category has two sub-categories that describe different bases for these experiences. Quotes from the focus groups describing the sub-categories are given in Table 5.

**Table 5:** Sub-categories and sample quotes from the focus groups^a^ describing the category “causes feelings of insecurity”

Category	Sub-category	Quotes
Causes feelings of insecurity	Lacks clarity	A2 (P1): But what kind of examination was it? No, they didn’t say. Just that it was a test for immunological defense. A2 (P3): You know, in this country, I’ve found after a while that we don’t get information from the authorities, we get it from each other. From friends, relatives, neighbors. But the country itself doesn’t give you any information … if we try hard, ask questions or what we’ve been exposed to, then you get to know, but not via them (the authorities). S1 (P2): It was hard to read the information we got on paper. First, the language is hard to understand. The time you put into trying to read is hard in itself … S3 (P2): I get a letter and I look at it and try to read. I can see and understand the date but I can’t understand anything more. S3 (P4): When I came to the doctor I started to say where it hurt. I couldn’t speak much Swedish then. They called in a Somalian man who couldn’t talk very good Swedish to help. He tried to explain what I said but didn’t succeed in saying everything to the doctor so well … it seemed like he (the interpreter) had said something completely different … the doctor took blood samples and wrote out medicines … I started to take the medicine … after about 2 or 3 days I got much worse. A3 (P3): I decline some things. She was there for half an hour and then I continued in English with her (the doctor) … there were some private things I didn’t want to say when she (the interpreter) was there.
	Does not give protection	A1 (P4): … the other thing is that the results when they come, the person knows what they are but the roommates don’t know. The person could have a dangerous disease. I had a roommate who went to another city for a few days and got letters that he asked me to read for him, and it turned out he had an inflamed liver … yes, and I don’t know about it … if I for example have something, you should know about it. A1 (P4): … there are certain cases that are psychological. There was a person who was crazy … with me in the same room. Yes, and he was left, he didn’t get any care … he gets letters now and then but we don’t know what he has. He might have a dangerous disease, he himself could hurt other people. Because he has epileptic seizures sometimes. A1 (P1): … no care is given for mental health. To be specific, the immigrant doesn’t find that mental care is adapted for the tragedy he comes from. S1 (P3): When they (the doctors) give a diagnosis they base it on what you’ve said yourself about your symptoms. And that’s not quite right. A4 (P3): I felt that it (the HEA) should have been given much earlier … I lived with a family, in a house with many family members. If I’d had a disease without visible symptoms it would have spread before the examination.

^a^*A* Arabic speaking focus group, *S* Somali speaking focus group, *P* Participant

### Lacks clarity

This sub-category describes the HEA as something that gives rise to feelings of insecurity owing to a lack of clarity in the communication before and during the HEA. This is experienced as being too brief to give a clear picture of what will happen during the examination. Feelings of insecurity are also triggered by the experience that the information is given without consideration to migrants’ limited knowledge about the Swedish society and to the information being given in an unfamiliar language. The lack of support from a qualified third bilingual person and poor trust in interpreters and the health service in general are experienced as leading to inadequate information, misunderstandings and unmet health needs.

### Does not give protection

This sub-category describes the experiences of HEA as something that causes insecurity as participants felt that it does not protect against other peoples’ diseases and mental health conditions that could be harmful. The HEA is not mandatory and is not given immediately upon arrival in Sweden, which means that infectious diseases from the home country or psychological difficulties caused by experiences of war and conflicts may affect others in the environment before the HEA takes place. As the HEA is mainly based on personal testimonies and does not focus on all types of health problems, it does not identify or protect against all possible expressed or unexpressed health problems. The fact that the results of the HEA are given individually means that there is no information about other migrants’ potentially harmful health conditions: it is thus not possible to be given protection from them. The absence of health-promoting information that is needed in the new environment, is experienced as decreasing the possibility to take care of one’s own health and take precautions against health risks. For example, about how to protect oneself from other refugees poor mental health and infectious diseases, and about common health issues in Sweden.

### Causes feelings of disappointment

This category describes experiences of the HEA as causing feelings of disappointment. It comprises two sub-categories that give different explanations for why the HEA is experienced as generating these feelings. Quotes from the focus groups describing the sub-categories are given in Table 6.

**Table 6:** Sub-categories and sample quotes from the focus groups^a^ describing the category “causes feelings of disappointment”

Category	Sub-category	Quotes
Causes feelings of disappointment	Does not fulfill the image of a health examination	A2 (P4): They say that it’s a general examination and then they should look at the whole body. Legs, nerves … everything. They just took blood and that was it. They should have a more thorough examination that they make, in different areas. A1 (P1): I thought that the person who came would get a complete examination. Like, we didn’t get x-rayed, what I know …even in Damascus, the wealthy people, we made a “check” every sixth months…you did an MRI, x-ray, blood tests, urine tests, stool tests, eyes and ears…That’s what the wealthy people did and then you knew exactly what you had. You got lung x-rays if you smoked if, god forbid, you had initial early signs of cancer or something. You understand? There were no x-rays here, not at all… A4 (P2): …when we went she asked for a whole hour about our health, what do you need, what sicknesses do you have? …I told her about the rash I got but she didn’t say we could look at it…But she didn’t ask to do tests right away so that we could be on the safe side.
	Does not focus on the individual	A1 (P1): The purpose isn’t to treat what there is they, they want to see if the person has diseases, maybe infections, spreading diseases like tuberculosis that affect society. S2 (P4): They don’t check what you want them to check. They wondered whether I had for example tuberculosis and AIDS. You should actually ask a person what worries they have and examine those parts. A2 (P1): …he said to them during the health examination that he felt unwell and needed a psychologist. They didn’t take it seriously…it wasn’t until today, seven months later that he got a time with a psychologist… S3 (P4): Yes, when I was at the doctor he asked me questions like that and that and that, have you heard that? Then I said no and the doctor was just quiet. A4 (P3): I got none (information) at all. She took information from me. She didn’t give me any advice…for example the most common illnesses here how I could protect myself or so, or about allergies that are common…if you get rashes or eczema from the air or the environment…In Syria you know what each season brings…But when we come here it was really that…when I took my daughter to the doctor today for her cough she said she wanted to examine her because right now there’s a lung infection going around in children…I didn’t have any idea about this, this illness. A1 (P1): They send (the results) only if you have a disease…a dangerous disease. That you have to follow up on. A2 (P1): But I think they should answer regardless of whether it’s positive or negative. Because you think the whole time that the letter got lost, or was sent to the wrong address, so you think about it until you get to know the results.

^a^*A* Arabic speaking focus group, *S* Somali speaking focus group, *P* Participant

### Does not fulfill the image of a health examination

This sub-category describes the HEA as something that causes feelings of disappointment as it does not fully correspond to the participant’s image of what a health examination should include or focus on. The concept of the HEA is based on information from various sources and from experiences of health examinations from the home country; it is not consistent with how the HEA is experienced in reality. It does not fulfill the image in that it does not identify all facets of ill health as there are only a limited number of tests. Further, there is no focus on mental health issues and no immediate help for all types of health problems but only acute problems.

### Does not focus on the individual

This sub-category describes the HEA as causing feelings of disappointment because it is not experienced that it is carried out for the benefit of the individual but rather in the interest of the security of society. These experiences come from the fact that the HEA does not show an interest in, act upon or follow up all health problems that are brought up by the individual or that are identified. There is also a pre-defined focus on certain specific diseases. The experience is that gathering information about the individual is more important than giving information about health and the test results.

## Discussion

The focus group discussions make it clear that participants have contradictory experiences of the HEA (health examination of asylum seekers), both between and within participants themselves; it is experienced as both beneficial and detrimental. The beneficial experiences of the HEA, described in the sub-categories “gives support or relief” and “cares on a personal level” are experienced to give some good. The harmful experiences of the HEA, described in the sub-categories “lacks clarity” and “does not give protection,” causes feelings of insecurity. The experiences of the HEA described in the sub-categories “does not fulfill the image of a health examination” and “does not focus on the individual” also cause feelings of disappointment.

The beneficial experience of the HEA in the form of its giving support and relief supports previous research showing participants’ appreciation of knowledge about their actual health status [22]. In our study, the participants had positive experiences of the HEA as they felt their health needs were met and they were supported in managing their health problems. The same views and experiences have been seen in several other studies. [7, 8, 22]. By providing support and inducing feelings of relief about participants’ health, the HEA can probably also contribute to a feeling of being cared for on a personal level. When there is an opportunity to take up and receive help with one’s own personal health issues, the chances are greater that the expectations of the HEA are achieved. The harmful experiences of the HEA support this too, when it is viewed as not focusing on the individual and thus not fulfilling the expectations of the purpose of the HEA.

The detrimental experiences of the HEA, described in the sub-categories “lacks clarity” and “does not fulfill the image,” mostly deal with the indistinct information and communication before, during and after the HEA. The results show, for example, that there are misunderstandings about the purpose and the content of the HEA as a result of disinformation caused by translation errors that occur when asylum seekers need help with translating information about the HEA, which is often given in Swedish. Language barriers also complicate the face-to-face communication between refugees and health care professionals during the HEA. Since a considerable proportion of newly arrived refugees are illiterate, have a low education and limited HL [9, 23], it should not be taken for granted that they have the ability to understand health information or use a dictionary or the internet to translate the information. It can also be difficult to find people who can translate properly [7, 22]. Being forced to ask relatives to translate personal health information, such as the results of an examination or test, may result in incorrect translations and violate a person’s integrity and prevent participants from speaking freely about health issues because they relate to stigma or shame [24]. The experience that the HEA did not meet with the participants’ expectations of both the content and procedure of a health examination, as well as the impression that the HEA is primarily of societal concern, might also lead to a hesitation to ask questions about individual health problems. This, together, with the fact that people with limited HL ask fewer questions during health encounters than those with higher HL [25], might contribute to limited knowledge of the HEA and health in general.

Lack of information, information that cannot be understood and misunderstandings are serious concerns as they may lead to inappropriate health decisions, missed opportunities for relevant care and, in the worst case, unnecessary ill-health. Specifically, the information being given in Swedish is not in agreement with the recommendations of the United Nations High Commissioner for Human Rights [26], where it is stated that health information should be available and understandable to those it concerns, that is, in one’s own preferred language. And while the Swedish guidelines for HEA [5] specifically state that the examination is voluntary and that this information must be given to the participants, their decision whether to participate may not be free in practice due to mistranslation and misunderstanding of the information.

That participants experienced feelings of disappointment in connection with the HEA is alarming considering previous research that reports that dissatisfaction with healthcare services can lead to a mistrust in healthcare [27] and negative future health-seeking behavior [28]. For many, the HEA is the first encounter with the healthcare system in the new country [7]. This might cause feelings of disappointment related to HEA that may influence the view of the entire healthcare system. This study and previous research [7, 29] also show that a common way to obtain health information among newly arrived refugees is to turn to more established fellow countrymen. Thus, the experiences of disappointment seen in our study may influence other asylum seekers in their choice of whether to participate in the HEA and may consequently affect their health conditions.

The view that the HEA does not give enough information about the healthcare system presented in the category “does not give protection” is in line with previous research [7], which highlights that the HEA is perceived as a missed opportunity for health communication about how the healthcare system works and how to navigate in it, but also that participants do not understand or trust the confidentiality of personal information within the health care system. This is remarkable, as a poor understanding of the healthcare system, for example, of where to go or how to navigate in the system, is a known barrier to healthcare among asylum seekers [28]. Research shows, for example, that newly arrived refugees with limited HL refrain from seeking care when they are in need of it, in comparison with those with higher HL [23]. This means that refugees have unnecessary ill-health and possibly encounter healthcare first when health problems have become very serious. Such delays may result in greater ill health and consequently higher costs if expensive care and treatment are required later.

Our interpretation is that the results of this study confirm previous ones [9, 23] showing that HL is of importance in the context of HEA. In this study we further gained knowledge that other experiences of HEA coexist and about why many experience that the communication does not work well. Several of the experiences seem to have to do with how the healthcare service organizes and communicates about the HEA. The study thus adds to our understanding that it is not only individual HL that matters for the experiences of HEA, but also HL on the organizational level. This is because many of the experiences of the HEA had to do with issues on the organizational level, which is the HL of healthcare organizations. The results indicate that organizational HL is of concern in how the participants experience the HEA, especially in terms of some of the ten described attributes [11] of health literate healthcare organizations (HLHO:s). According to those, such organizations shall, for example: use HL strategies in interpersonal communication and confirm understanding at points of contact; provide easy access to health information and services and navigation assistance; and design and distribute printed, audiovisual and social media content that is easy to understand and act upon. Two other attributes that are relevant for the HEA are that the organization shall train its healthcare staff to be health literate, and have a leadership that integrates health literacy into its mission, structure, and operations.

The results indicate that attributes of HLHOs are sometimes met, for example by “giving support and relief” and “care on a personal level.” For example, facilitation of two-way communication by using interpreters and offering participants the possibility to ask questions are related to the HLHO attribute requiring implementation of HL strategies in interpersonal communication [11]. Support is also experienced by receiving help with health problems that are found, and by receiving information about self-management and how living habits impact health, which can increase participants HL. At the same time, the results show that the attributes are sometimes not met, for example, by “lacking clarity”, “not giving protection” and “not focusing on the individual.” The experiences in all three categories, to some extent have to do with misunderstandings of what HEA is and how the healthcare system works, caused by inappropriate and insufficient information. According to the defining features of an HLHO, it should be easy to gain access to health information, health services and navigation assistance [11]. Given that previous research shows that the majority of newly arrived refugees in Sweden have limited HL [9, 23], while the results of this study show that organizations are not always health literate, greater effort is needed to secure that all organizations offering an HEA take HL into account in their work. When administered by an HLHO, an HEA would play an important role in improving asylum seekers´ HL and, by extension, their health. If measures aiming at creating an HLHO are not taken, those with limited HL, who statistically also have the poorest health [30], will continue to benefit least from an HEA, thus preserving health on unequal terms.

The study has strengths and limitations. The fact that the participants had experiences from 15 different centers that offer HEAs in different parts of Sweden ensures wider representation and is not judged to influence the trustworthiness of the results. The heterogeneity among the participants in total, in the form of experiences from different centers offering HEA and in terms of gender, country of origin, age and education, contributed to a variation of experiences and a richness of content, which appears in the results in the form of the broadness of content in the categories. As the aim was to explore participants’ experiences of the HEA, it was appropriate to employ a method in which the essential feature was discussion among the participants. The homogeneity within each focus group in terms of focus groups consisting of people with the same gender and education level is judged to contribute to an openness in the discussions, giving rich and evocative information. The bilingual moderators’ culture competence meant that they would have an adequate understanding of what the participants talked about [19] and could identify topics that would be important to discuss in more depth. However, focus group discussions in the participants’ native languages also made it difficult for the researcher to control the discussions. Probing questions asked directly by the researcher might have led to more detailed knowledge about some issues. The use of an interpreter had other drawbacks as well [19], such as being able to gather less information overall because of the communication between three people. The use of female moderators seemed not to hinder the discussions, but other more sensitive information may have been disclosed if a male had moderated the focus groups in which men participated. We did not aim to investigate differences in findings by gender, country of origin or education level as these questions were beyond the scope of the present study, and also would require special considerations in recruitment and interviews of participants as well as focus group moderation.

The results of our study may be transferable and useful for improving health services that target both refugees and migrants in general. The results suggests that an organization may work on the individual level, by improving information and communication to patients, but also by reducing health literacy on the organizational level, for example by developing HL interpersonal communication strategies. The fact that the discussions were translated into Swedish before the analysis began means that the expressions from each participant had gone through an interpretation process, which may influence the reasonableness and the accuracy of the research. However, several measures were taken to reduce possible interpreter bias [19]: uniform guides for translation and transcription of the audio files were given out, 10% of each transcript was assessed by a third bilingual person and moderators gave feedback on the final results. Cross-cultural research has its limitations [19], for example in terms of collecting and analyzing data when the researchers do not speak the same language as the study population. However, by collecting data in the refugees’ native languages and using as few people as possible in the translation of the data, it was possible to obtain comprehensive information from a group that is otherwise often excluded from research.

### Clinical implications

On the basis of the results of this study and research in the field of HL, we believe that the following actions can improve HEA and lead to better experiences of it among participants. The information and communication in and about the HEA need to be better, clearer, more comprehensive and match participants‘ needs and prerequisites. It is advised that information and communication with the participants should be carried out in their native language and that qualified, experienced interpreters or cultural mediators are used in the communication before and during the HEA. To verify that the information is understandable, the “Teach-back” [31] method can be used, in which the recipients of information, in their own words, repeat the information they just received to the person who gave the information. The Askme3 [32] method, which encourages patients and families to ask three specific questions at the encounter to better understand their health condition and what they need to do to stay healthy, can be used to help participants ask questions.

As to the content of the HEA, the participants may have more positive experiences if it is more comprehensive, better responds to the participants’ own needs, mental problems and chronic diseases, and refers the participants to other health care institutions and ensures that they receive help within a reasonable period of time. It would also be good to give a longer period for the examination in order to allow time for the participants’ own health concerns and the extra time it takes to communicate through an interpreter. This and more health-promoting and preventive health information about how the healthcare is organized and can be accessed would probably also reduce the participants‘ feelings about the HEA being more for society than for the individual. Further, these measures might increase the asylum-seekers’ HL and their trust in the healthcare system, which in turn might lead them to use the healthcare system in a more efficient way in the future. Better supervision to ensure that the HEA is implemented in a similar way in all parts of the country, while at the same time being person-centered and considering the participants‘ own health problems, would be good and is necessary for following the principle of care on equal terms. However, changing the content and the allotted time might be difficult owing to politics and priorities in the healthcare system. Any disappointment about the content may also be reduced by clear communication to increase knowledge and accurate information about the HEA. This can affect the risk of incorrect expectations about what it contains and what a participant can get help with. Finally, the HEA should be offered earlier, immediately when asylum seekers arrive in the country. This may cause more people to feel motivated to participate in it, and reduce the fear of being infected or hurt by other new arrivals during the first period in the country.

## Conclusions

In this study, the HEA was experienced as beneficial and detrimental at the same time. It was viewed as beneficial by giving support or relief and care on a personal level and detrimental by lacking certain aspects, not giving protection, causing feelings of insecurity, not fulfilling the image of a health examination or not focusing on the individual. The feelings were influenced by the experiences of information and communication before, during and after the examination and how health literate the organizations providing the HEA are. To achieve more satisfied participants, it is crucial that all organizations providing an HEA become health literate and person-centered.

## Additional file

Additional file 1: Interview guide. (DOCX 24 kb)

## Data Availability

The datasets generated and/or analyzed during the current study are not publicly available due the qualitative nature of our project. Personal narratives can more readily be associated with individual respondents. Data may be made available upon reasonable request. Contact person is Josefin Wångdahl: josefin.wangdahl@pubcare.uu.se.

## Abbreviations

HEA: Health examination for asylum seekers
HL: Health literacy
HLHO: Health literate healthcare organizations
