# Supplementary material for: Perspectives on health examination for asylum seekers in relation to health literacy – focus group discussions with Arabic and Somali speaking participants
Source: BMC Health Serv Res. 2019 Sep 18;19:676. doi: 10.1186/s12913-019-4484-4 (PMC6751618; doi:10.1186/s12913-019-4484-4)
Supplement: Supplementary file 1 — Interview guide. (DOCX 24 kb) [file 12913_2019_4484_MOESM1_ESM.docx]

**Interview guide**

What is your name, where do you come from and where did you do the health examination for asylum seekers?

**The health examination for asylum seekers**

**Initial question about the health examination**

- You who are here have all participated in a health examination for asylum seekers. What are your thoughts about the health examination?

**Communication and information in connection with the health examination**

- How well did the communication work when you participated in the health examination for asylum seekers?
- How could communication be improved?
- How did you experience the information you received when you participated in the health examination?
- In what way did you benefit from that information?
- What information do you think was missing?

**The benefit of the health examination**

- In general, in what way did you benefit from the health examination?
- If you think freely, what could be done to increase the benefits participants get from the health examination?

**Health literacy**

In order to improve and take care of one's health, it is useful to know certain things. Being able to obtain, understand and use information about health, health care and various diseases are some examples of useful skills.

**Access**

- Consider the time before the health examination for asylum seekers: what did you do to find information about the health examination?
- How do you obtain information about health in general?

**Understand**

Consider the time around the health examination for asylum seekers: what did you feel about your ability to understand the information you got about the health examination and the information you got during the health examination?

What did you feel about understanding health information in general?

**Appraise**

Consider your health examination for asylum seekers: what did you do to assess whether the information you received was accurate and could be trusted?

- How do you assess whether health information can be trusted in general?

**Apply**

- If you think about the information you received in connection with the health examination, in what ways have you used it?
- How do you use health information in general?
- If you think freely, how could a health examination be set up so that communication and information work perfectly? So that everyone involved understands what is being said, can talk about their health problems, ask questions and get answers to the questions they ask.

**Final questions**

Through our conversations we have received very important information from you that will be used for improving the health examinations. During this hour we have talked about communication and information in connection with the health examination for asylum seekers, as well as the usefulness of participating in it. In addition, we have talked about various abilities that may be important in order to take care of and improve one’s health.

- Is my summary of today's conversation correct or is there something I have misunderstood?

- Do you have something to add?
